# Questions about Residual Cell Viability in Cryopreserved Human Amniotic Membrane and Its Impact on Clinical Applications

**DOI:** 10.3390/biomedicines10102456

**Published:** 2022-10-01

**Authors:** Camille Gaudet, Lauriana Solecki, Bastien Mathéaud, Stephane Odet, Christophe Meyer, Aurélien Louvrier, Florelle Gindraux

**Affiliations:** 1 Service de Chirurgie Maxillo-Faciale, Stomatologie et Odontologie Hospitalière, CHU Besançon, F-25000 Besançon, France; 2 Service d’Ophtalmologie, CHU Besançon, F-25000 Besançon, France; 3 Laboratoire de Nanomédecine, Imagerie, Thérapeutique EA 4662, Université Bourgogne Franche-Comté, F-25000 Besançon, France; 4 INSERM, EFS BFC, UMR1098, University Bourgogne Franche-Comté, RIGHT Interactions Greffon-Hôte-Tumeur/Ingénierie Cellulaire et Génique, F-25000 Besançon, France

**Keywords:** viability, cell survival, amniotic membrane, matrix, cryocongelation, cryopreservation, differentiation, quality controls

## Abstract

We questioned the relevance of evaluating residual cell viability in human amniotic membrane (hAM) after its cryopreservation since cell survival is controversial and its ability to act as a matrix (including the presence of growth factors and cytokines) appears to be most important for tissue regeneration purposes. We also discussed the usefulness of osteodifferentiating amniotic cells in whole hAM for bone repair applications. We have evidence that determining residual cell viability after cryopreservation and hAM osteodifferentiation is not justified.

## 1. Introduction

Given the topic you proposed in “Advanced Research in Stem Cells and Regenerative Medicine” related to perinatal derivatives, we would like to highlight a redundant question about cell viability in human amniotic membrane (hAM) and its potential impact on tissue regeneration. Indeed, residual cell survival in fresh and/or cryopreserved hAM is controversial. The need to also quantify/qualify cell viability was a requirement. Consequently, improved cell survival and its impact on regeneration are disputable. In this context, we also question the need for amniotic cell differentiation in whole hAM for bone repair. 

## 2. Human Amniotic Membrane

For many years, hAM has been widely used to treat ocular surface disorders and to improve wound healing [1,2,3,4]. Oral and maxillofacial surgeons have also started to use hAM in various indications, such as mucosal defects, guided bone regeneration, root coverage of gingival recession, mandibular vestibuloplasty, oronasal fistulae management and bisphosphonate-related osteonecrosis of the jaw [5,6,7]. 

hAM is composed of a single layer of amniotic epithelial cells (AECs), a basement membrane containing amniotic mesenchymal stromal cells (AMSCs), and an avascular stroma, underlaid by the chorion [8]. Its therapeutic effects are mainly due to the release of growth factors and cytokines. 

Banking of hAM started in 1966 [9]. Currently, the usual storage formats are cryopreserved, lyophilized or air-dried [10]. 

hAM has been used clinically in many other indications, such as in covers/bandages or implanted materials [11,12]. Even if it is an allograft, little to no immunogenicity has been reported after implantation of fresh hAM [1,2,4,5,13,14,15], frozen hAM [4] or cryopreserved hAM [11,16,17].

To date, ophthalmology is one of the most routine applications of hAM. In France, the cryopreserved format has been used since 2000 [18]. With 5349 hAM patches distributed over the last 12 years for the treatment of several ocular pathologies, it has been suggested that the ability of hAM to promote epithelium healing may stem from the basement membrane’s tendency to facilitate epithelial cell migration, promote epithelial differentiation and reduce inflammation, scarring and vascularization [19]. 

## 3. Cell Death/Survival and Differentiation 

The presence of residual viable cells after the cryopreservation process is controversial [8,20,21] and depends on the technique used [22,23,24]. In a previous study using the EZ4U assay, there was a significant 60% decrease in the viability of amniotic cells isolated from hAM cultured in a specific MSC medium and from cryopreserved hAM compared to the fresh format [21]. Additionally, cell death was confirmed by Trypan blue and Calcein-AM/DAPI staining performed on whole hAM and on cells derived from it. 

Concerned about cell viability, we wondered if trypan blue staining on intact tissue, like is performed with cornea grafts [25], would be applicable to hAM. We concluded that trypan blue staining combined with Giemsa staining is a useful additional step in the quality controls necessary for the release of grafts, as it is easy to implement in a tissue bank [21]. 

We assessed the possibility of hAM osteodifferentiation by culturing whole tissue as previously suggested by Lindenmair [26]. In this line, patches of whole hAM and amniotic cells (hAMSCs and hAECs) isolated from fresh hAM were cultured for 3 weeks in two different osteogenic media or in control medium usually used for MSC expansion [27]. All conditions (fresh or cultured hAM; intact or hAM-derived cells) were tested for phenotypic and functional analyses with standard approaches (cell culture and staining, histological and immunolabelling) as well as original approaches (tissue staining, energy-dispersive X-ray and X-ray diffraction). We showed that—in osteogenic conditions—hAECs surprisingly had a mesenchymal phenotype with osteocyte function, and even hydroxyapatite synthesis, suggesting the osteogenic potential was mainly focused on this epithelial layer. Our results are concordant with works performed on hAECs isolated from the different hAM regions showing a heterogeneous cell population with different pluripotency and proliferation marker expressions (octamer-binding transcription factor 4 (OCT-4), tyrosine protein kinase KIT (c-KIT), sex-determining region Y-box 2 (SOX-2), a-fetoprotein, cyclic AMP response element-binding (CREB) protein and the phosphorylated active form of CREB (p-CREB)), proliferative ability and osteogenic potential [28]. 

All together, we assumed that in vitro pre-osteodifferentiation of hAM did not appear to be necessary for bone repair because native hAM already had an innate pre-osteoblastic potential. We demonstrated that fresh and osteodifferentiated hAM had similar in vivo tissue degradation, suggesting that in vitro hAM pre-osteodifferentiation did not influence its in vivo biocompatibility [29]. At that time, we were convinced that cell viability for osteodifferentiation purposes in bone repair was a requirement, and we agreed that hAECs had the highest stemness potential/characteristic compatible with this indication. 

In a collaboration with Fenelon and colleagues, no statistical difference between fresh versus cryopreserved hAM was found [20], with hAMSCs survival in hAM presumably being more resistant to the cryopreservation process. In a critical-sized calvarial bone defect in mice—without graft material—cryopreserved hAM induced more bone formation when the mesenchymal layer covered the defect compared to when the defect was left empty; fresh hAM was not superior. Given these findings, we strongly questioned the role of cell survival. Additional studies performed with decellularized and/or lyophilized hAM have highlighted a matrix role for hAM rather than a cellular role in guided bone regeneration or substitution of the induced membrane technique [30,31,32,33].

Additionally, some studies have shown that cells or hAM itself from amniotic subregions differ considerably in their morphological and structural properties and the content/release of certain bioactive factors [28,34,35,36]. Our current working hypothesis is that cell viability could also vary by hAM region, another reason why its quantification/qualification is ambiguous.

## 4. Potential Impact of Cell Viability When hAM Is Used as a Matrix

It has been suggested that the best cryopreservation and storage methods should depend on the hAM’s intended application [37,38,39,40]. Although some authors have sought to improve cell viability [24], based on the excellent clinical outcomes in several ocular pathologies, matrix integrity seems to be more important than epithelium viability to the hAM’s biological properties [19].

Along these lines, some authors have recently reported that after two freeze–thaw cycles, continued cell viability is not expected in hAM preparations used for ophthalmological purposes and therefore does not need to be evaluated [41].

Similarly, Lamon et al. reported that for ocular surface reconstruction, hAM is used as a biological dressing and related trophic growth factors. For these reasons, basement membrane integrity and stromal preservation are critical, while no hAM-derived cells are expected to repopulate and replicate on the recipient eye [42]. Therefore, they did not investigate cell viability or quantify DNA levels before and after cryopreservation (Leal-Marin et al. 2020).

## 5. Conclusions

Until now, residual cell viability in cryopreserved hAM has not been taken into consideration during clinical use. We have evidence that cell differentiation in the whole hAM seems to have limited impact in the bone repair context. 

Over 4 years, the French Biomedicine Agency has recorded 12,137 hAM transplants. A single biovigilance incident was declared in 2020 because of the graft’s size and poor quality without causing any adverse event. In addition, out of 5349 distributed hAM products, no adverse reactions or other unexpected events were reported by surgeons after a 12-year follow-up [19]. 

Outside the ophthalmology context, like Ragazzo et al. [43], we did not see any signs of inflammation when cryopreserved hAM was used for the management of medication-related osteonecrosis of the jaw [44].

Considering the absence of data about potential immunogenicity with cryopreserved hAM and the debate about cell survival after cryopreservation, we recognize that its major function is to serve as a matrix, which means that verifying residual cell viability after cryopreservation is pointless. Additionally, there is no clear evidence for the endogenous role of amniotic cells from cryopreserved hAM in tissue repair. As a result, the previous statement that no hAM-derived cells are expected to repopulate and replicate on the recipient eye can be extrapolated to oral mucosa, and therefore, hAM can be said to act mainly as a matrix (containing growth factors and cytokines) in this indication. 
